# Whole blood expression profiling from the TREAT trial: insights for the pathogenesis of polyarticular juvenile idiopathic arthritis

**DOI:** 10.1186/s13075-016-1059-1

**Published:** 2016-07-07

**Authors:** Kaiyu Jiang, Laiping Wong, Ashley D. Sawle, M. Barton Frank, Yanmin Chen, Carol A. Wallace, James N. Jarvis

**Affiliations:** Department of Pediatrics, University at Buffalo Jacobs School of Medicine and Biomedical Sciences, Clinical & Translational Research Center, 875 Ellicott St., Buffalo, NY USA; Irving Cancer institute, Columbia University College of Physicians and Surgeons, 1130 Saint Nicholas Ave., New York, NY 10032 USA; Oklahoma Medical Research Foundation, Arthritis & Clinical Immunology Program, 800 NE 13th St., Oklahoma City, OK 73104 USA; Division of Rheumatology, Seattle Children’s Hospital and Research Institute, 4800 Sand Point Way NE, MA.7.110, Seattle, WA 98105 USA; Genetics, Genomics, and Bioinformatics Program, University at Buffalo Jacobs School of Medicine and Biomedical Sciences, Buffalo, NY USA

**Keywords:** Juvenile idiopathic arthritis, Microarray, Whole blood, Gene expression, Pathogenesis

## Abstract

**Background:**

The Trial of Early Aggressive Therapy in Juvenile Idiopathic Arthritis (TREAT trial) was accompanied by a once-in-a-generation sample collection for translational research. In this paper, we report the results of whole blood gene expression analyses and genomic data-mining designed to cast light on the immunopathogenesis of polyarticular juvenile idiopathic arthritis (JIA).

**Methods:**

TREAT samples and samples from an independent cohort were analyzed on Affymetrix microarrays and compared to healthy controls. Data from the independent cohort were used to validate the TREAT data. Pathways analysis was used to characterize gene expression profiles. Furthermore, we correlated differential gene expression with new information about functional regulatory elements within the genome to develop models of aberrant gene expression in JIA.

**Results:**

There was a strong concordance in gene expression between TREAT samples and the independent cohort. In addition, rheumatoid factor (RF)-positive and RF-negative patients showed only small differences on whole blood expression profiles. Analysis of the combined samples showed 158 genes represented by 176 probes that showed differential expression between TREAT subjects at baseline and healthy controls. None of the differentially expressed genes were encoded within linkage disequilibrium blocks containing single nucleotide polymorphisms known to be associated with risk for JIA. Functional analysis of these genes showed functional associations with multiple processes associated with innate and adaptive immunity, and appeared to reflect overall suppression of STAT1–3/interferon response factor-mediated pathways.

**Conclusions:**

Despite their limitations, whole blood expression profiles clearly distinguish children with polyarticular JIA from healthy controls. Whole blood expression profiles identify several immunologic pathways of biologic relevance that will need to be pursued in homogeneous cell populations in order to clarify mechanisms of pathogenesis.

**Trial registration:**

ClinicalTrials.gov registry #NCT00443430, originally registered 2 March 2007 and last updated 30 May 2013.

## Background

The Trial of Early Aggressive Therapy in Juvenile Idiopathic Arthritis (TREAT; ClinicalTrials.gov registry #NCT00443430) was an National Institute of Health (NIH) funded clinical trial [1] that compared two therapeutic regimens for treatment of newly-diagnosed polyarticular juvenile idiopathic arthritis (JIA). One treatment arm used methotrexate (MTX) as an initial therapy, while the other used a combined regimen of MTX, the tumor necrosis factor (TNF) inhibitor etanercept (ET), and oral prednisone. As part of the TREAT trial, whole blood was collected for RNA expression studies at specific time points during the course of the first year of therapy. Using whole blood expression data from the TREAT subjects, we have previously reported on the feasibility of developing expression-based prognostic biomarkers for children with the polyarticular form of JIA [2]. However, the whole blood gene expression data also provide a window through which we might also gain valuable insights into both the pathogenesis of JIA and the underlying biology of treatment response, both of which are currently poorly understood.

While whole blood (and buffy coat) expression data are inherently “noisy” (among other things, they reflect gene expressions in multiple cells and cell subsets), there are both technical [3, 4] and computational approaches [5] that can be used to improve the signal-to-noise ratio in whole blood expression data and derive meaningful mechanistic insights. Furthermore, projects like the NIH Encyclopedia of Functional DNA Elements (ENCODE) and Roadmap Epigenomics have provided investigators with a wealth of information from which to derive mechanistic insights from gene expression data. In this study, we used whole blood gene expression data derived from baseline samples from children enrolled in the TREAT study, coupled with data-mining from public resources, to identify novel pathways that contribute to JIA disease pathogenesis.

## Methods

### Patient samples

We have previously described the TREAT baseline samples [2]. Patients entered the TREAT trial between October 2007 and November 2009 [1]. All children fit international criteria for polyarticular-onset JIA [6]. Parents of these children gave informed written consent to provide samples for translational uses, and the protocol to use these specimens was approved by the TREAT study oversight committee and the University of Oklahoma Institutional Review Board. The patients included 19 boys and 45 girls, aged 2–14 years. ANA were detected in 30 of the girls and 10 of the boys. Approximately 2.5 ml of blood was collected at the time of enrollment (month 0) prior to treatment in a PAXgene tube (PreAnalytiX GmbH, Hilden, Germany). Samples were stored at –80 °C. A summary of patient ages and characteristics is shown in Table 1.

**Table 1:** Phenotypic characteristics of patients at month 0 (baseline)

	Arm 1	Arm 2
Female	19 (63.3 %)	26 (76.5 %)
Male	11 (36.7 %)	8 (23.5 %)
Aged 2–6 years	7	5
Aged 7–11 years	11	15
Aged 12–16 years	12	14
Age (years), mean ± SD	10.5 ± 4.5	11.4 ± 3.6
RF-positive	9 (30 %)	12 (35.3 %)
ANA-positive	22 (73.3 %)	18 (52.9 %)^a^

Values are given as *n* (%) unless otherwise indicated

^a^One missing value

*ANA* Antinuclear antibody, *RF* rheumatoid factor, *SD* standard deviation

### Healthy control samples

Controls consisted of 8 healthy girls and 11 healthy boys between the ages of 7 and 13 years recruited from the OU Children’s Physicians General Pediatrics clinic. The protocol for obtaining these specimens was approved by the University of Oklahoma IRB (#13205). Anesthesia for the phlebotomy was provided using topical lidocaine/prilocaine solution. These samples are hereafter referred to as healthy children (HC).

### Independent cohort

In addition to the above, whole blood PAXgene specimens were obtained from an independent cohort of children with newly diagnosed, polyarticular-onset JIA recruited from the University of Oklahoma Health Sciences Center pediatric rheumatology clinic. These children ranged in age from 3 years to 15 years and consisted of 4 boys and 6 girls. All patients in this cohort were rheumatoid factor (RF)-negative. These samples are hereafter referred to as the Oklahoma cohort (OK). Table 2 summarizes the characteristics of these patients.

**Table 2:** Characteristics of JIA patients from the Oklahoma cohort

Patient number	Age	Sex	RF status	ANA status
1	9 years	F	Negative	Negative
2	12 years	F	Negative	Negative
3	9 years	M	Negative	Negative
4	11 years	F	Negative	Positive
5	7 years	F	Negative	Negative
6	3 years	F	Negative	Positive
7	8 years	M	Negative	Negative
8	14 years	M	Negative	Negative
9	15 years	F	Negative	Positive
10	4 years	M	Negative	Negative

*ANA* Antinuclear antibody, *F* female, *JIA* juvenile idiopathic arthritis, *M* male, *RF* rheumatoid factor

All research procedures were carried out strictly following the IRB-approved protocols.

### RNA processing

RNA was purified from whole blood PAXgene specimens using a PAXgene Blood RNA kit (Qiagen, Valencia, CA, USA) as recommended by the manufacturer, including a DNAse (Qiagen) step to remove genomic DNA. Globin transcripts, which reduce labeling efficiency of whole blood cell RNA and decrease signal-to-noise ratios on microarrays [7], were reduced using GLOBINclear-Human (Ambion, Austin, TX, USA). Final RNA preparations were suspended in RNase-free water, quantified spectrophotometrically, and analyzed for RNA integrity by capillary gel electrophoresis (Agilent 2100 Bioanalyzer; Agilent Technologies, Palo Alto, CA, USA).

### Microarray analysis

cRNA was produced from reverse transcribed cDNA using the Illumina® TotalPrep RNA Amplification Kit (Ambion, Inc., Austin, TX, USA), hybridized to Illumina WG-6 v3 or Illumina HT-12 v4 human whole genome microarrays, and stained according to the manufacturer’s directions. Array hybridizations were undertaken in three separate batches. The first batch consisted of the 19 healthy controls and 26 baseline samples hybridized on Illumina WG-6 v3 arrays. The second batch consisted of the remaining patient samples hybridized to Illumina HT-12 v4 arrays. The independent cohort of OK samples were hybridized on Illumina WG-6 v3 slides. Gene microarray data have been made available to the scientific public (GEO Accession Number GSE55319).

### Statistical analysis

All statistical analyses were carried out in R (www.r-project.org). To facilitate statistical analyses relative to healthy controls, it was necessary to combine data from different array batches. Due to the difference in the arrays it was necessary to create combined datasets using only those probes that were present on both array formats. Illumina probe IDs were used to identify 39,426 common probes. Datasets were variance stabilized and normalized using robust spline normalization via the *lumi* package [8, 9]. Prior to statistical analysis non-responding probes were filtered out of the datasets using the detection *p* value provided by the Illumina quality control metrics to eliminate probes not responding at higher than background levels.

Differential gene expression analysis was performed using the *limma* package [10, 11]. The false discovery rate (FDR) was estimated using the method described by Benjamini and Hochberg [11]. Statistical significance of gene expression was determined at FDR ≤0.05. Gene lists of interest were exported from R and uploaded to Ingenuity IPA (Ingenuity Systems, Inc., Redwood City, USA) for further functional analysis.

#### Network analyses of differentially expressed genes from whole blood expression profiles

We used the Ingenuity Pathway Analysis (IPA) software (Ingenuity Systems®, Redwood City, CA, USA; IPA Summer Release, June, 2015) for network analysis, with differentially expressed (DE) genes as input following the default setting of a maximum 35 genes per network. IPA network generation transforms the query genes into a set of relevant networks based on its extensive, curated Ingenuity Pathway Knowledge Base database [12, 13]. IPA utilizes a multi-stage heuristic algorithm in constructing networks through an iterative process that optimizes both interconnectivity and number of query genes under the constraint of network size. Briefly, IPA constructs networks using gene connectivity with other genes under the assumption that the gene with the highest number of connections is the most important and, thus, has the most influence. These connections represent regulatory interactions that may be either direct (e.g., the protein product of gene “A” directly regulates the expression of gene “B”) or indirect (genes “A” and “B” are regulated by the same transcription factor). The last step of IPA network construction is score calculation using the Fisher Exact test on a hypergeometric distribution. The Fisher Exact test in IPA defines the null hypothesis as being a similar proportion of query genes map to a network in the same proportion as the entire reference gene set map to the network. A network score is derived from a *p* value (score = –log10 *p* value) that indicates the probability of the query genes (defined as target molecules in IPA) in a network being randomly associated within a connecting network. It is important to note that the network score does not infer network quality; it simply indicates the fitness between a reference network and the network of query genes.

#### Linking genetic and expression data

The recent completion of a genome-wide fine mapping study for JIA [14] extends the list of previously known disease-associated genetic variants [15] and provided us with the opportunity to determine whether there is a genetic linkage to gene expression in JIA. Using the bedtools program [16] we intersected DE genes identified in the comparison between JIA and HC with linkage disequilibrium (LD) blocks of JIA-associated single nucleotide polymorphisms (SNPs) extracted from the JIA fine mapping study [14] as well as other known regions of risk as reviewed by Hersh and Prahalad [15]. We obtained LD block information from the SNAP database (http://www.broadinstitute.org/mpg/snap) using the 1000 genome project pilot1 and HapMap3 with a cutoff of *r*^2^ < 0.9 and a distance limit of 500 bases.

## Results

### Correlation of TREAT samples with the Oklahoma cohort

From the TREAT study we analyzed 44 baseline samples from 28 RF-negative patients and 16 RF-positive patients, plus 19 control samples from healthy children. In addition, we analyzed independent samples from 10 RF-negative patients (the OK cohort). Oklahoma and TREAT data were first normalized without using the COMBAT algorithm that we previously applied to the TREAT data to filter out batch effects [2], as there is only one condition (baseline) in the OK subjects for comparison and, thus, the algorithm cannot differentiate between biological variation and technical variation (batch effects). Our assumption was that batch effects would weaken the statistical correlation relative to the true biological situation, and any correlation we might find using this approach would thus be significant.

**Fig. 1:**
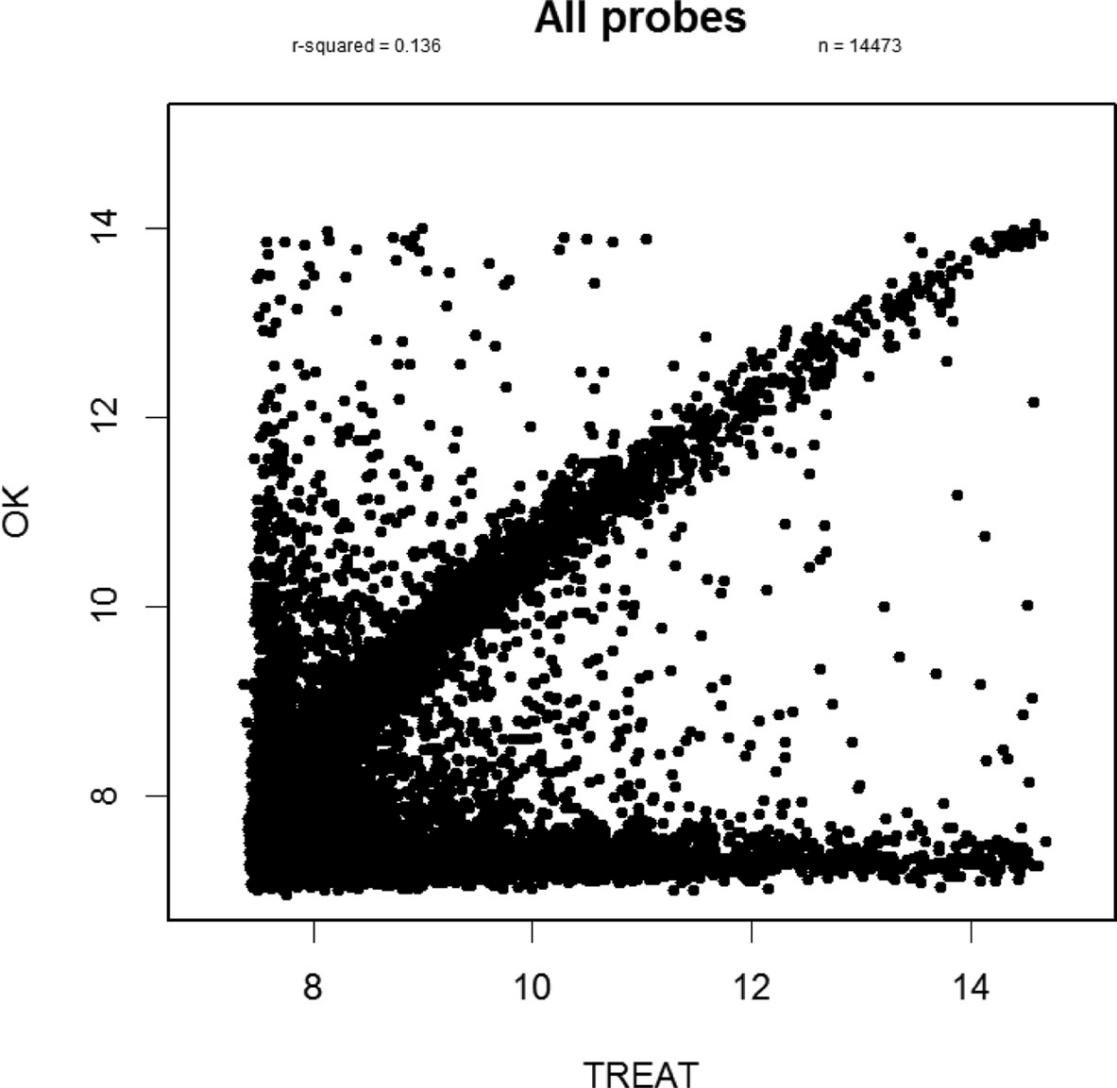
Correlation between gene expression of probes between Oklahama (*OK*) and TREAT data. Four observations are shown; first, at x = y, probes correlate well; second, with y < 8, probes with low expression in the Oklahoma data but a range of expression in the TREAT data; third, when x < 8, probes with low expression in the TREAT data but a range of expression in the Oklahoma data; and, lastly, random scatters of probes in between Oklahoma and TREAT data

We first created a scatter plot correlating the OK and TREAT expression values for all probes (Fig. 1). As shown in the figure, there are four distinct groups of probes: 1) probes that appeared to match well (roughly, x = y); 2) probes with low expression in the Oklahoma cohort but demonstrating a range of expression in TREAT subjects (seen along the bottom of the graph); 3) probes that showed low expression in the TREAT subjects but a range of expression values in the Oklahoma patients (a small number running up the left-hand side of the plot); and 4) a small scattering of probes in between.

**Fig. 2:**
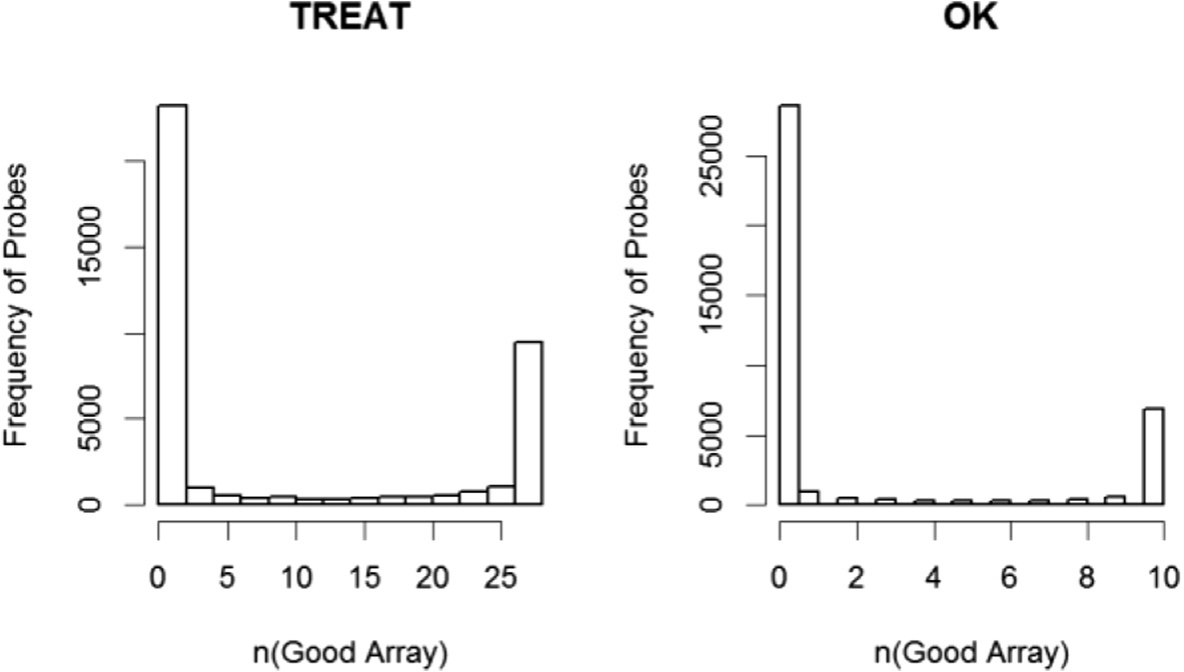
Distribution of probe qualities in TREAT data (*left*) and Oklahoma data (*OK*; *right*)

We then asked whether those probes that are low in one set but high in the other may reflect poor quality in the arrays, especially for low-expression probes. To investigate this possibility, we used Illumina array GenomeStudio software, which calculates and reports a detection *p* value representing the confidence that a given transcript is expressed above the background defined by negative control probes. The cut-off we used for trimming probes from the arrays prior to differential expression analysis was to only select probes with *p* ≤ 0.01. The histograms in Fig. 2 show the distribution of the probe qualities in the two datasets. For each probe we counted the number of arrays on which it had a detection *p* value ≤0.01. Using this approach, we identified multiple poor quality probes on the both array sets.

**Fig. 3:**
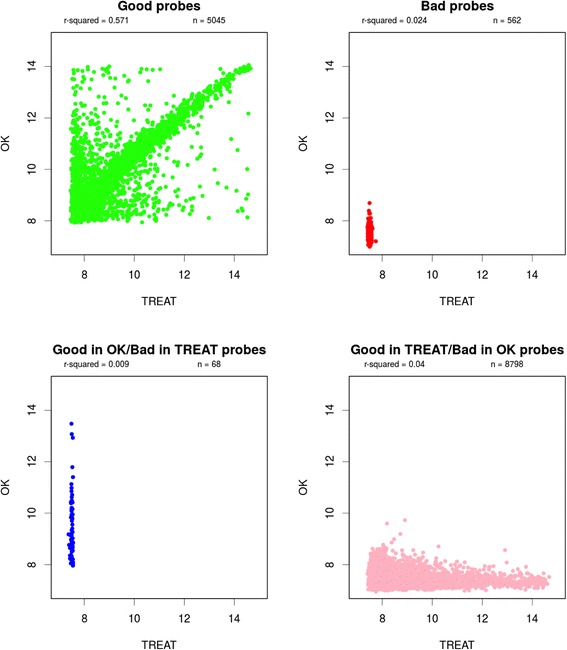
Correlation of probes between TREAT and Oklahoma (*OK*) for (*top left*) good probes with good quality in both datasets and (*top right*) bad quality probes in both datasets. *Bottom left*, good probes in TREAT but bad in Oklahoma; *bottom right*, good probes in Oklahoma but bad in TREAT. Using probes that are high quality in both datasets improves correlation between the two datasets

Upon splitting the probes into groups based on their quality, we discerned that there were technical issues creating the two unmatched tails. In the comparison, we required that all 10 of the Oklahoma arrays be good quality (Fig. 3). Using this approach, we found that good probes (top left) displayed good quality in both datasets and bad quality probes were bad in both. Selecting only probes that are high quality in both datasets significantly improves the *r*^2^ (0.627 for our two datasets).

We also note that in our biomarker paper [2] we were able to corroborate gene expression in the TREAT and Oklahoma cohorts using quantitative polymerase chain reaction (qPCR) [11]. Having assured ourselves of the reproducibility of the array results, we proceeded to data analysis.

### Differential gene expression

We first compared RF-positive (*n* = 16) and RF-negative (*n* = 28) samples. We found only 7 genes represented on 8 probes that showed differential expression between these groups; these genes are shown in Table 3. These results are consistent with our previous observation that RF-positive and RF-negative samples group together when analyzed through hierarchical cluster analyses [2]. For this reason, we determined that it would be reasonable to analyze all 44 samples (RF-positive and RF-negative) as a group.

**Table 3:** Differentially expressed genes in juvenile idiopathic arthritis between RF-positive and RF-negative patients

Probe_ID	Symbol	Mean expression RF^–^ (Log2)	Mean expression RF^+^ (Log2)	Fold change (Log2)	FDR
ILMN_1690443	C14orf82	8.775	9.284	–0.509	0.046
ILMN_1717594	DKFZp761E198	8.567	9.061	–0.493	0.028
ILMN_1721113	HLA-C	9.43	10.126	–0.696	0.04
ILMN_1778202	FLJ40722	11.76	12.259	–0.499	0.032
ILMN_1806165	HSPA6	10.943	11.429	–0.486	0.036
ILMN_1912662		11.38	11.896	–0.516	0.038
ILMN_2243516	C11orf63	10.335	10.939	–0.604	0.036
ILMN_2285713	TDP1	10.931	11.421	–0.49	0.015

*FDR* false discovery rate, *RF* ^–^ rheumatoid factor negative, *RF* ^+^ rheumatoid factor positive

**Fig. 4:**
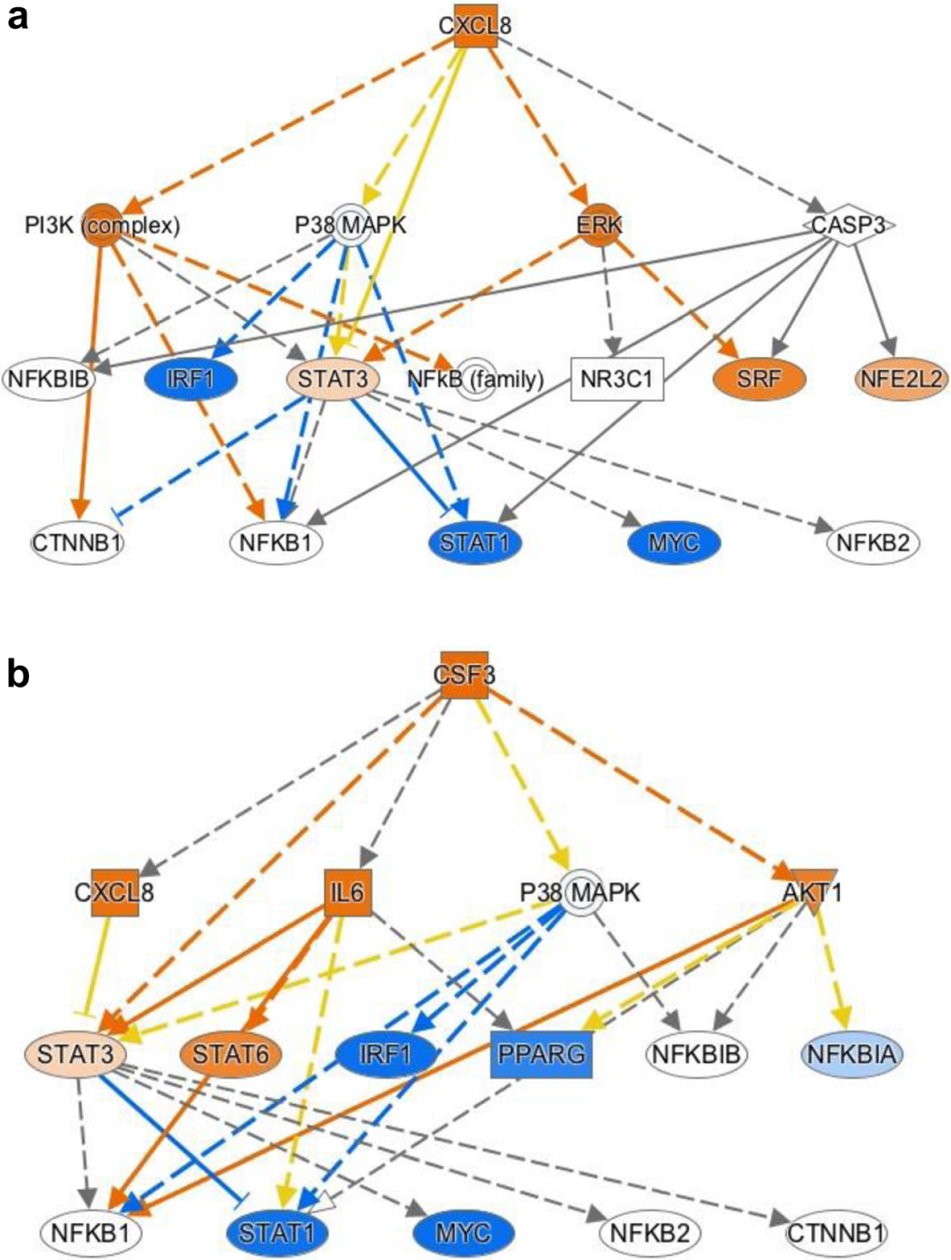
Mechanistic network derived from differential gene expression analysis of upstream regulators of differentially expressed genes using IPA and comparing untreated to healthy controls. Nodes in *orange* reflect predicted activation, while those in *blue* are predicted to be inhibited based on the patterns of differential gene expression. Upstream regulators CXCL8 (**a**) and CSF3 (**b**) show an activation pattern

There were 158 genes represented by 176 probes that showed differential expression with at least a 1.4-fold difference, with an FDR of 0.05, when TREAT study subjects were compared with healthy control children. The analysis of these genes using the Ingenuity database showed particular enrichment for genes regulating leukocyte adhesion and extravasation. This pattern is reflected in the network shown in Fig. 4a, demonstrating a pattern of interleukin (IL)-8 CXCL8 activation. IL-8 is produced by multiple leukocyte subsets, including macrophages [17, 18], as well as other cell types such as endothelial cells [19]. IL-8 acts through multiple G protein-coupled receptors and is a potent chemoattractant for neutrophils. It is interesting to note that we have recently demonstrated that G protein-coupled receptor signaling networks showed extensive re-organization when TREAT study subjects responded to therapy [5]. Patterns of gene expression reflect regulation by CSF3, which, like IL-8, is a potent activator of neutrophils (Fig. 4b). We have previously shown that neutrophils in JIA show aberrant patterns of activation that are linked to the metabolic pathways through which neutrophils produce myeloperoxidase and superoxide ion [20].

**Fig. 5:**
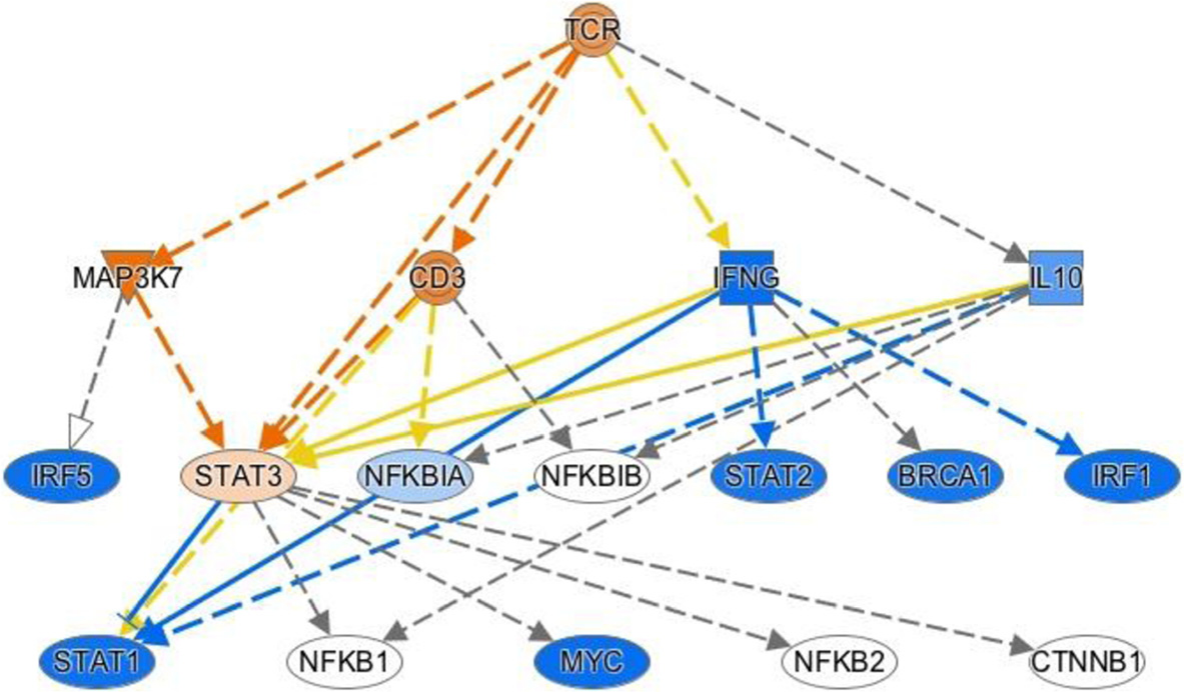
Mechanistic network derived from differential gene expression analysis of upstream regulators of differentially expressed genes using IPA and comparing untreated JIA to healthy controls. Nodes in *orange* reflect predicted activation, while those in *blue* are predicted to be inhibited based on the patterns of differential gene expression. CD3–T-cell receptor (*TCR*) activation is predicted from the pattern of gene expression

The baseline TREAT expression data also reflect activation of adaptive immune responses through CD3–T-cell receptor-mediated signaling, as shown in Fig. 5. T cells have long been accepted as mediators of JIA pathogenesis [21], and thus this finding was expected.

**Fig. 6:**
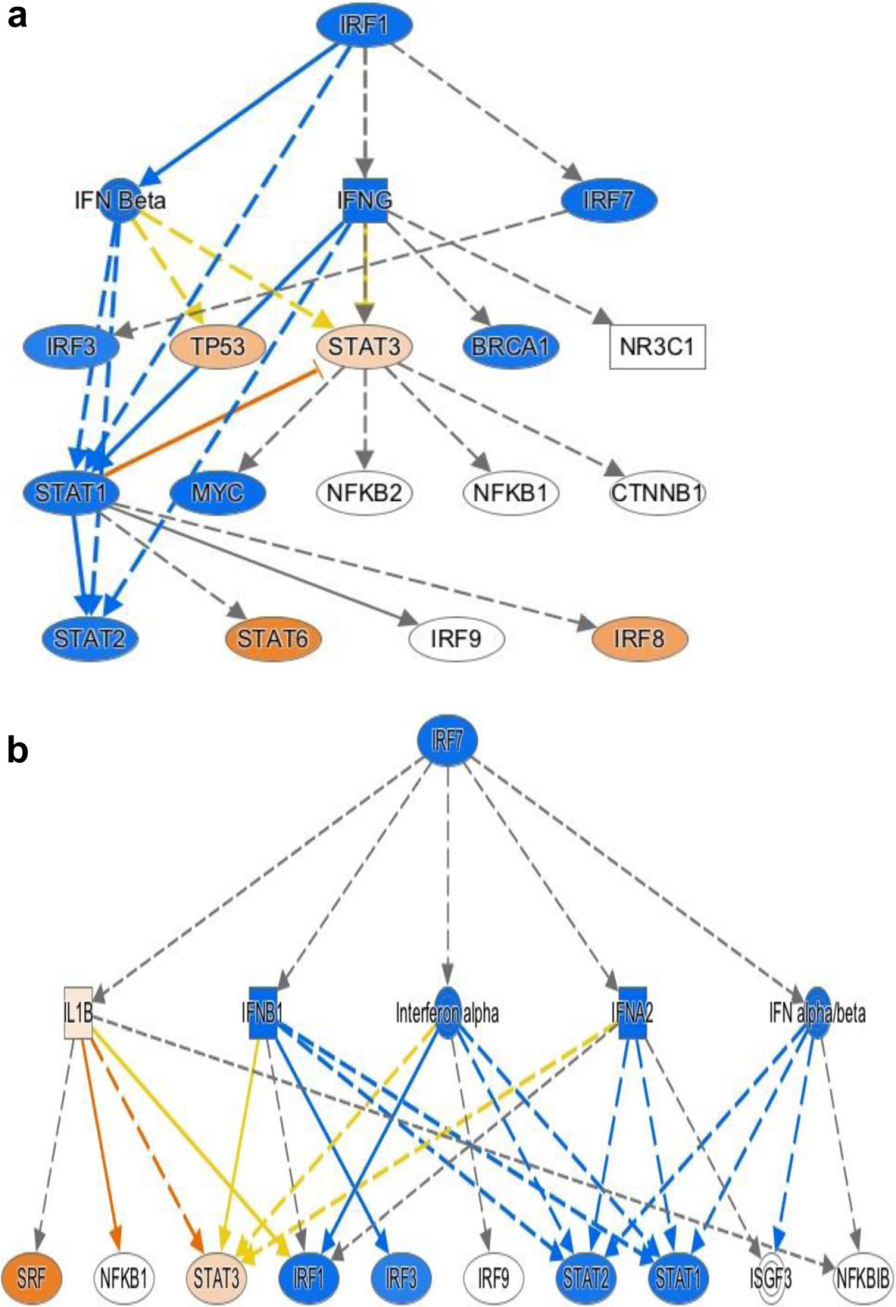
Mechanistic network derived from differential gene expression analysis using IPA and comparing untreated JIA to healthy controls. Nodes in *orange* reflect predicted activation, while those in *blue* are predicted to be inhibited based on the patterns of differential gene expression. Suppression of IRF1 (**a**) and IRF7 (**b**) regulated networks is predicted from this analysis

**Fig. 7:**
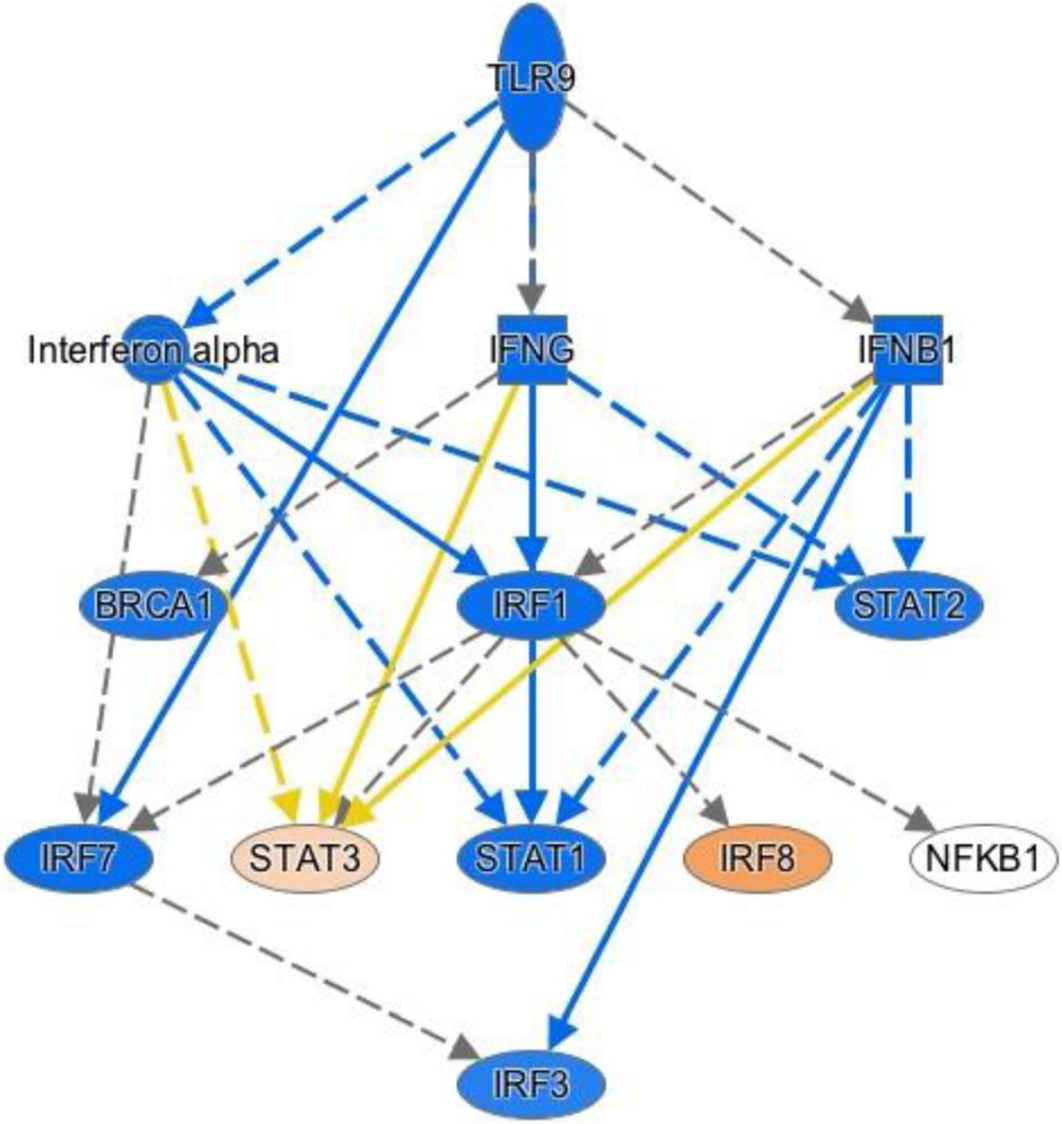
Mechanistic network derived from differential gene expression analysis using IPA and comparing untreated JIA to healthy controls. Nodes in *orange* reflect predicted activation, while those in *blue* are predicted to be inhibited based on the patterns of differential gene expression. Suppression of TLR9-regulated networks is predicted from this analysis

It is interesting to note that, at the same time, the expression data simultaneously reflect a pattern that we have previously identified from RNA sequencing data in human neutrophils [22]: an apparent suppression of interferon response factor (IRF)-mediated pathways leading to a suppression of type1 and type 2 interferons, as shown in Fig. 6. This pattern of suppression reflects a broader suppression of Toll-like receptor (TLR)9 activation, as shown in Fig. 7. The TREAT expression data suggest a previously unrecognized role for transforming growth factor (TGF)B1 in JIA, although its role in adult rheumatoid disease has been long recognized [23], and we have shown that TGFB1 is overexpressed in children with active JIA on therapy compared with children who have achieved clinical remission on medication [24]. These findings suggest aberrant patterns of activation and/or gene regulation within the adaptive immune system.

### Linking genetic and expression data

The search for overlaps between the DE genes identified in the comparison between healthy controls and baseline TREAT subjects and LD blocks containing JIA-associated SNPs yielded no DE genes within any of the observed LD blocks. We next determined the closest LD blocks to the DE genes, and identified 75 LD blocks containing JIA-associated SNPs situated near 38 DE genes, with distances ranging between 345 and 390 kilobases. Next, we interrogated the upstream regulators of the DE genes as identified on Ingenuity analysis, i.e., IRF1, IRF3, IRF5, STAT1, STAT2, and STAT3. We queried the LD blocks containing the JIA-associated SNPs using the bedtools program. We downloaded ENCODE transcription factor binding site (TFBS) data from UCSC Genome Browser ENCODE data at http://hgdownload.cse.ucsc.edu/goldenPath/hg19/encodeDCC/wgEncodeRegTfbsClustered/and extracted TFBSs of the regulators of interest. Intersection analysis demonstrated that 53 LD blocks containing JIA-associated SNPs overlapped 232 TF binding sites for the regulators of interest. We applied Fisher’s Exact test for enrichment analysis of TF binding sites overlapping with the LD blocks containing JIA-associated SNPs using all ENCODE TF binding sites as background. This analysis showed no statistically significant evidence for enrichment of IRF1, IRF3, IRF5, STAT1, STAT2, or STAT3 binding within the LD blocks containing JIA-associated SNPs (Fisher’s Exact test *p* value 0.091).

## Discussion

In this paper, we analyzed the TREAT whole blood gene microarray data in an attempt to gather insights into disease mechanisms in polyarticular JIA. We found that the whole blood expression profiles reflect complex interactions between innate and adaptive immunity, a finding that is consistent with our previous reports that both peripheral blood mononuclear cell (PBMC) [24] and neutrophil [25] gene expression profiles are abnormal in untreated children with JIA. We were not able to answer the question of whether the innate or adaptive immune aberrations are primary. While an increasing body of data shows that neutrophils are important mediators of adaptive immune responses [26–31], it is equally plausible that the transcriptional aberrations we see in neutrophils are the result of an altered cytokine milieu generated from altered T-cell function. In support of the idea that the neutrophil defect is a primary aspect of the disease is our finding that neutrophil gene expression profiles in JIA patients remain distinctly abnormal even after PBMC profiles begin to resemble those of healthy children in response to therapy [32].

These findings are also significant for what they do not tell us. Among the 158 genes that showed differential expression between baseline TREAT samples and those of healthy controls, none was located within LD blocks where there is known genetic risk for JIA [14, 15], nor are the identified LD blocks enriched for binding sites for the TFs that the whole blood expression data suggest may be important regulators of the differentially expressed genes. This finding corroborates published work demonstrating that most of the genetic risk for JIA lies within the non-coding genome [14]. We have recently demonstrated that most of the regions identified by Hinks et al. [14] are enriched (above genome background) for H3K4me1/H3K27ac-marked enhancers that can be identified in both neutrophils and CD4+ T cells [33]. Thus, if polyarticular JIA, like many complex diseases, can be characterized by the presence of so-called expression quantitative trait loci (eQTL) [34], it seems likely that the loci that most strongly influence expression will be located in non-promoter regulatory regions (e.g., enhancers, insulators, and so forth) and reflect complex layers of transcriptional control rather than perturbed function of the protein products of specific genes.

Our findings here suggest potentially useful targets for therapy in JIA. For example, the broad suppression of type 1 and type 2 interferon responses in JIA may reflect overall suppression of TLR9-mediated processes. TLR9 is an intracellular pattern recognition receptor that detects highly methylated DNA, which is common in bacterial and viral genomes (and relatively rare in mammalian genomes) [35]. In recent years, TLR9 has become an attractive therapeutic target for immune modulation in immune diseases [36, 37], as well as cancer [38–42]. Given these findings, it is hardly surprising that hydroxychloroquine, which suppresses TLR9 pathways [43, 44], has been shown to be ineffective in JIA. The TREAT whole blood expression data suggest that strategies to augment TLR9 responses might be more promising.

There are obviously limitations to these data and their interpretation. The first is the inherent “noisiness” of whole blood expression profiles. Whole blood expression profiles represent an amalgam of peripheral blood cells, including abundant leukocyte subtypes such as neutrophils and platelets, and less abundant cells such as monocytes, natural killer (NK) cells, and even circulating CD34+ cells [45]. Furthermore, while the complete blood counts of the TREAT baseline subjects did not deviate from the range typically seen in children of the same age, it is possible that the differences in expression profiles reflect expansion of small leukocyte subsets not typically identified on complete blood counts performed in a standard clinical laboratory. The noisiness and relative insensitivity of whole blood expression profiling can be reduced by removing globin genes before the RNA labeling step [3, 46], as we did here, but this step reduces only a small portion of the complexity that limits the utility of whole blood expression data. Leukocyte subset transcriptomes show a considerable degree of specificity, reflecting the specific immunologic functions of each cell type. Thus, while there are considerable commonalities in the transcriptomes and regulatory regions of peripheral blood leukocytes [47–49], it is likely that there are critical elements of leukocyte function/dysfunction in polyarticular JIA (e.g., B cells, monocytes, Th17 cells) that simply cannot be identified on whole blood expression profiling. Thus, while whole blood or blood leukocyte expression profiling has been invaluable in allowing us to develop a mechanistic understanding of significant pathologic disturbances, such as sepsis or blunt trauma [50], it seems likely that complex functional genomics approaches of specific leukocyte subsets will be required to fully elucidate the pathogenesis of more subtle phenotypes where inflammation is chronic and more indolent, as is the case in polyarticular JIA [22].

## Conclusions

Whole genome expression profiling of untreated children with polyarticular JIA reveals complex transcriptional differences when compared with healthy controls. Activation of leukocyte chemotaxis/extravasation pathways and neutrophil activation by CSF3 are reflected in whole blood transcription analyses. At the same time, suppression of STAT1–3/IRF pathways, as we have previously reported in JIA neutrophils [22], is revealed in the whole blood expression profile. None of the genes that showed differential expression between children with JIA and healthy control children is encoded within LD blocks containing known JIA-associated SNPs. These findings suggest that genetic risk loci for JIA either exert their effects before the disease phenotype emerges or involves more subtle and complex layers of transcriptional regulation (e.g., by *trans*-acting enhancers [33]) than can be discerned from whole blood expression profiles.

## Abbreviations

DE, differentially expressed; ET, etanercept; FDR, false discovery rate, HC, healthy children; IL, interleukin; IPA, Ingenuity Pathway Analysis; IRF, interferon response factor; JIA, juvenile idiopathic arthritis; LD, linkage disequilibrium; MTX, methotrexate; NIH, National Institute of Health; OK, independent Oklahoma cohort; PBMC, peripheral blood mononuclear cell; RF, rheumatoid factor; SNP, single nucleotide polymorphism; TFBS, transcription factor binding site; TGF, transforming growth factor; TLR, Toll-like receptor; TREAT, Trial of Early Aggressive Therapy in Juvenile Idiopathic Arthritis
